# Accessory Lobe of Liver: A Rare Hepatic Variation With Diagnostic Dilemma

**DOI:** 10.7759/cureus.34020

**Published:** 2023-01-20

**Authors:** A Bhagyashri, Praisy Joy, Manisha R Gaikwad, Sanjukta Sahoo

**Affiliations:** 1 Anatomy, All India Institute of Medical Sciences, Bhubaneswar, Bhubaneswar, IND

**Keywords:** hepatic diverticulum, couinaud's segment, accessory groove, accessory lobe, liver

## Abstract

The liver is a very dynamic organ. Still, the gross anomalies of the liver are sparse. The accessory lobe of the liver is a rare anatomical variation with a prevalence of less than 1%. We present a case of an accessory lobe and two accessory grooves of the liver in a cadaver. The accessory lobe was an isolated anomaly, sessile in presentation, and attached to normal liver parenchyma. Although accessory lobes are rare, knowledge about them will reiterate to surgeons and radiologists to be mindful of them and to avoid misdiagnosis.

## Introduction

The liver, wedge-shaped largest abdominal viscera which occupies a substantial portion of the right hypochondrium and even spreads into the left hypochondrium up to the left axillary line, and is segmented into right, left, caudate and quadrate lobes based on the peritoneal attachments externally [1,2]. The liver, being a very dynamic organ, undergoes rapid growth from infancy to adulthood, but gross anomalies of the liver are rare.

Although accessory lobes of the liver are less known compared to vasculobiliary variations, a good understanding of the classical anatomy as well as potential anatomic variations is crucial for successful liver operations [3]. The prevalence of this variation is considered to be less than 1% and this congenital ectopic hepatic tissue is most commonly caused by embryonic heteroplasia but it can also be an acquired one which is even very rare due to trauma or surgery [4].

## Case presentation

**Figure 1:**
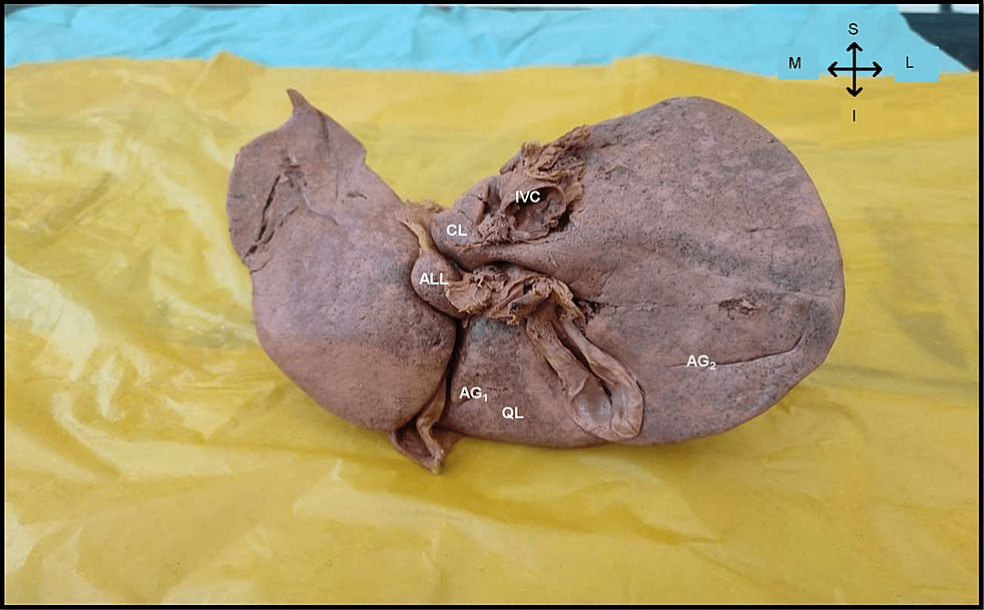
Posterior view of the liver showing the accessory lobe and accessory grooves. ALL – Accessory lobe of liver; AG1 – Accessory groove in quadrate lobe; AG2 – Accessory groove between colic and renal impression; QL – Quadrate lobe; CL – Caudate lobe; IVC – Inferior vena cava.

**Figure 2:**
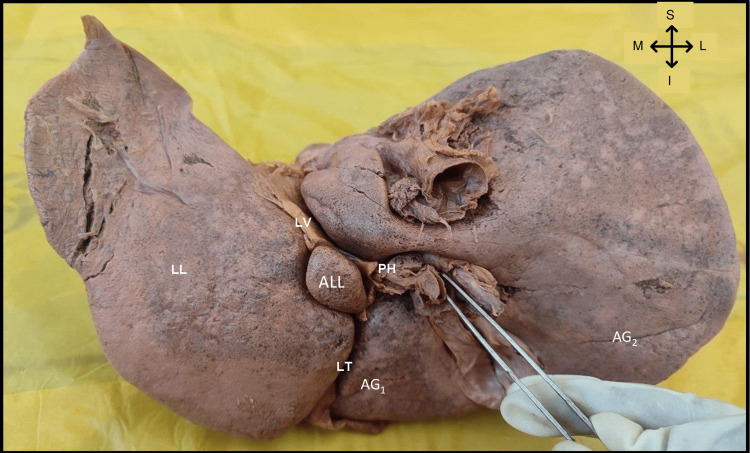
Posterior view of the liver showing the relations of accessory lobe of liver. PH – Porta hepatis, LV – Ligamentum venosum (Lateral); LL – Left lobe of liver (Medial); LT – Ligamentum teres (Inferior); ALL – Accessory lobe of liver; AG1 – Accessory groove in quadrate lobe; AG2 – Accessory groove between colic and renal impression.

As part of routine dissection classes, during detailed observation and demonstration of the liver for first-year undergraduate students at the Department of Anatomy, we noticed a very rare anatomical variant of the liver, an accessory lobe of the liver. It was located between the I and III Couinaud segments (Figure 1). The whole liver was 1.25 kg. The accessory lobe of the liver was 3.7 cm x 1.7 cm x 1.6 cm. Ligamentum venosum and Porta Hepatis were lateral and the whole of the left lobe of the liver was medial relations to the accessory lobe. The inferior relation was ligamentum teres (Figure 2). Apart from the accessory lobes, two accessory grooves were also noted one in the right lobe between the colic and renal impression and the other in the quadrate lobe (Figure 1).

## Discussion

Most congenital malformations related to organs require knowledge of their embryological genesis in order to properly define them, accessory lobe of the liver is a supernumerary liver lobe with normal hepatic parenchyma which is in continuity with the normal liver tissue [4,5]. These are usually incidental findings during routine dissection, autopsy, or during surgical procedures [6].

During the fourth week of intrauterine development, the hepatobiliary system develops from the hepatic diverticulum - a ventral outgrowth from the distal part of the foregut [7]. According to previous literature, presently there are two hypotheses regarding the development of the accessory lobe of the liver, one theory claims that during early stages of development, the embryonic liver curls outwards and forms an accessory lobe while other claims that an accessory lobe develops as a result of increased intra-abdominal pressure by the rapidly developing liver [8,9].

Collan et al. classified the accessory lobe of the liver into four types - a large accessory liver lobe connected to the main liver by a connective tissue strand; a small accessory liver lobe connected to the main liver, weighing 10-30g; an ectopic liver that is not in contact with the liver proper and is primarily attached to the gallbladder or intra-abdominal ligaments; and a microscopic liver ectopic found in the gallbladder wall [10]. There are similar classifications by Gurba et al. and Tancredi et al. based on the weight and volume of the accessory lobe of the liver [11,12].

Stattaus et al. have given an alternative classification of the accessory lobe of the liver with clinical implications, into pedunculated and sessile one [13]. In the present case, the accessory lobe of the liver is a small lobe attached to the normal liver parenchyma and it was a sessile lobe.

Pedunculated lobes can undergo torsion which results in infarction and at certain times they can act as points of bleeding also, but this is very rare, and the patient presents with acute abdomen and there are reported cases of malignant transformation of this accessory lobe of the liver. And sometimes they can be misdiagnosed as an enlarged lymph node or a tumor since they vary in location [14].

Accessory hepatic fissures can mimic pathological liver nodules on CT, and they can also act as points of implantation for peritoneally disseminated tumors [2,5,14]. Accessory hepatic lobes are presumed to be asymptomatic since they are most commonly an incidental finding or seen in cadavers, but sometimes they are associated with congenital anomalies in the pediatric population. Ito et al., Elmasalme et al., Grunz et al., Sanguesa et al., and Koplewitz et al. have described about the association of omphalocele and accessory lobe of the liver in the pediatric population. The age of diagnosis was between one day to 14 years for the children, the possible cause than be the increased intra-abdominal pressure. Azmy et al. have described about the association of the accessory lobe of the liver in a child with Beckwith-Wiedemann Syndrome. Bladder exstrophy, umbilical hernia and renal agenesis was described by Ladurner et al. [15]. Woldeyes in his case report has mentioned the co-existence of undescended testis in a cadaver with the accessory lobe of the liver [4]

## Conclusions

Accessory lobes of the liver are rare anatomical variations. Most commonly it is an incidental finding during autopsy or laparotomy. Accessory lobes can present as acute or recurrent right upper quadrant pain. It is a diagnostic dilemma for enlarged periportal lymph nodes and hepatocellular carcinoma. Knowledge about the existence of the accessory lobe of the liver is essential for planning safer surgical procedures.
